# QuickStats

**Published:** 2014-07-11

**Authors:** FROM THE NATIONAL CENTER FOR HEALTH STATISTICS

**Figure:**
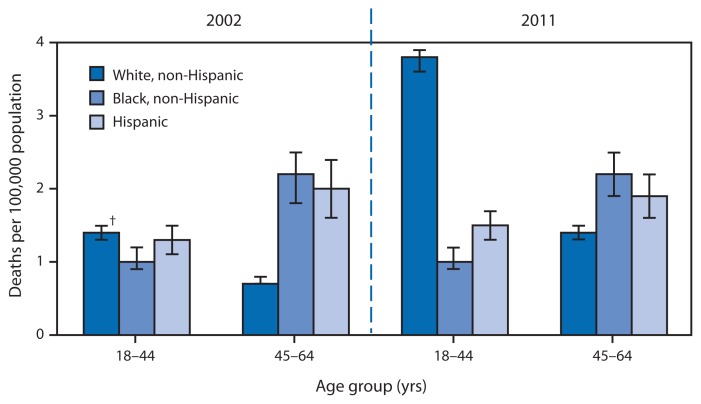
Rates of Drug Poisoning Deaths Involving Heroin,* by Selected Age and Racial/Ethnic Groups — United States, 2002 and 2011 * Per 100,000 population. Based on *International Classification of Diseases, 10th Revision* underlying cause codes X40–X44, X60–X64, X85 and Y10–Y14, with a multiple cause of death code of T40.1 (heroin). ^†^ 95% confidence interval.

In the decade from 2002 to 2011, the annual number of drug poisoning deaths involving heroin doubled, from 2,089 deaths in 2002 to 4,397 deaths in 2011. In 2002, non-Hispanic blacks aged 45–64 years and Hispanics aged 45–64 years had the highest rates of drug poisoning deaths involving heroin (2.2 and 2.0 deaths per 100,000, respectively). In comparison, in 2011, non-Hispanic whites aged 18–44 years had the highest rate. From 2002 to 2011, the rate for non-Hispanic whites more than doubled for the 18–44 years age group (from 1.4 to 3.8 deaths per 100,000) and doubled for the 45–64 years age group (from 0.7 to 1.4 per 100,000). The rates for both age groups of Hispanics and non-Hispanic blacks did not significantly change during the decade.

**Source:** National Vital Statistics System mortality data. Available at http://www.cdc.gov/nchs/deaths.htm.

**Reported by:** Holly Hedegaard, MD, hdh6@cdc.gov, 301-458-4460; Li-Hui Chen, PhD; Margaret Warner, PhD.

